# Attempted suicide of ethnic minority girls with a Caribbean and Cape Verdean background: rates and risk factors

**DOI:** 10.1186/s12888-017-1585-7

**Published:** 2018-01-17

**Authors:** Diana D. van Bergen, Merijn Eikelenboom, Petra P. van de Looij-Jansen

**Affiliations:** 1 Research Unit for Youth Studies, Department of Education, University of Groningen, Groningen, The Netherlands; 2 Department of Psychiatry and the Amsterdam Public Health research institute, VU University Medical Center Amsterdam / GGZ inGeest, Amsterdam, The Netherlands; 3 Department of Research and Business Intelligence, Municipality of Rotterdam, PO BOX 1130, 3000 BC Rotterdam, The Netherlands

## Abstract

**Background:**

WHO data shows that female immigrants in Europe attempt suicide at higher rates than ‘native’ women and ‘native’ and immigrant men. Empirical studies addressing attempted suicide of female immigrants of Caribbean (Antillean-Dutch and Creole-Surinamese-Dutch) as well as Cape Verdean descent in Europe are however scarce. We aim to increase knowledge about rates and risk factors of girls of Caribbean and Cape Verdean descent living in the Netherlands.

**Methods:**

We conducted logistic regression on a dataset that consisted of self-reported health and well-being surveys filled out by 5611 female students, age 14–16, in Rotterdam, the Netherlands (Antillean Dutch *N* = 357, Creole-Surinamese-Dutch *N* = 130, and Cape Verdean-Dutch *N* = 402, and Dutch ‘natives’ *N* = 4691). We studied if girls of these minority groups had elevated risk for attempted suicide. Risk indicators that were suspected to play a role were investigated i.e. household composition, socio-economic class, externalizing problems, emotional problems and sexual abuse.

**Results:**

We found that rates of attempted suicide among Antillean (14%), Creole-Surinamese young women (15.4%) were higher than of ‘native’ Dutch girls (9.1%), while rates of Cape-Verdean girls (8.3%) were rather similar to those of ‘native’ girls. Not living with two biological parents was a risk factor for ‘native’ girls, but not for girls of Caribbean and Cape Verdean descent. Emotional problems and sexual abuse seems to be a risk indicator for suicidality across all ethnicities. Aggressive behaviour was a risk factor for Antillean Dutch and ‘native’ girls.

**Conclusions:**

Our findings underscore the need for developing suicide prevention programs for minority girls in multicultural cities in western Europe, in particular those of Caribbean descent. Results suggest the importance of addressing socio-economic class and educational background for suicide prevention, which bear particular relevance for Caribbean populations. Referral in the case of sexual trauma and low psychological wellbeing seems critical for reducing suicidal behaviour in girls, regardless of ethnicity.

## Background

### Attempted suicide in young women and the relationship with ethnicity

Attempted suicide in adolescence is an important concern for public health [1]. Knowledge about attempted suicide of youth in Europe is mostly guided by studies among its majority (‘white’) populations. However demographic trends show that the number of ethnic minorities in Europe is increasing. Specific immigrant and ethnic minority groups in Europe [2] are at increased risk for attempting suicide, a finding also observed for the USA [3].

Attempted suicide is more often found among females than males [4]. Especially the period of mid adolescence (14–16 years), shows a peak in the risk for attempted suicide for females health [1]. Considering an increased risk for attempting suicide exists for (some) ethnic minority groups on the one hand, and among young females on the other hand, a heightened risk of suicidal behavior could be expected for minority females. Underpinning this assumption, females from Turkish descent in Germany, Switzerland and the Netherlands and females of South Asian descent in the United Kingdom and The Netherlands attempt suicide at higher rates than ‘native’ women and ‘native’ and immigrant men [2]. Additionally, girls with a Hispanic background in the USA demonstrate disproportionate suicide risk [3]. Thus, young females of certain ethnic minority groups seem to be a vulnerable group with regard to suicidal behavior in Europe and in the USA. However, there is an incomplete picture on rates of and risk factors for attempted suicide in immigrant female populations in Europe at present.

There are indicators that young women of African and Caribbean descent in the west are at risk for suicidal behavior. Young Caribbean and ‘Black’ British women aged 16 to 34 had the highest rates of suicide attempts of all ethnicities in the United Kingdom [5]. In the Netherlands, Creole-Surinamese women were shown to have increased suicide rates [6]. In the US, ‘black’ young females have an increased risk of attempting suicide resulting in medical treatment, compared to white young US women [3]. Therefore, the present study aims to investigate the rates and risks for suicidal behavior of young women of Caribbean and (mixed) African descent in the Netherlands, that is, Caribbean Dutch (Antillean and Creole-Surinamese) girls and Cape-Verdan Dutch girls.

### Migration history of Caribbean and Cape-Verdean immigrants in the Netherlands

Caribbean-Dutch constitute the second largest immigrant group in the Netherlands (500.000 people). The first wave of Antillean and Surinamese migrants to the Netherlands consisted of people who came for educational purposes in the 1950’s and 1960’s. During the eighties and nineties, Antillean migration to the Netherlands rapidly increased, especially by economically deprived individuals. Among the Surinamese, a second large migration wave occurred in the late seventies, just after the country gained its independence from the Netherlands [7]. While Cape Verdean immigrants are a relatively small immigrant group in the Netherlands (20.000 persons), the city of Rotterdam (The Netherlands) is however host to the second largest community of Cape Verdeans in Europe. The harbor of Rotterdam had a central function for the sea trade in the 1970’s where many immigrant Cape Verdeans sought and found jobs, started families, and continued to reside in The Netherlands.

### Risk factors for attempted suicide among girls of Caribbean and Cape-Verdean descent

Attempted suicide in adolescence is best understood as an interplay between socio-economic dimensions, family, individual and socio-cultural factors [8]. An immigrant status often coincides with a low socio-economic position, which influences the wellbeing of immigrant children, including the risk of attempting suicide [8]. Many Creole-Surinamese, Antillean and Cape Verdean families in the Netherlands have been found to have minimal financial resources compared to majority Dutch families.

Next, risks for attempting suicide may also exist in relation to the family structure. In Caribbean as well as Cape Verdean cultures, the family is traditionally shaped within a matrifocal system, in which the upbringing of children is *a joint venture* among female (extended) family members and the (biological) father’s role is considered to be marginal [9]. Many researchers argue that the matrifocal system is a non-problematic future of Caribbean and African societies [9]. However, upon migration, this may change, since the support system that used to surround the mother has often eroded. Thus, Caribbean and African family households may then start resembling single-parent families ‘western style’, for which there is evidence for risks of suicidal behavior amongst children [10].

On the individual level, both internalizing (emotional) and externalizing problems [11]. independently seem to enhance the propensity to attempt suicide. Emotional problems often coincide with feelings of hopelessness and depressed mood that precedes suicidal behaviour. In Europe, ‘Black’ British female adolescents [12] as well as Antillean Dutch girls [13] were found to have higher scores of emotional disorders compared to ‘native’ girls. Surinamese Dutch girls did not differ much from majority females [13] (no information available on Cape Verdan girls). Furthermore, externalizing problems were reported among black female adolescent European populations at a higher rate than ‘natives’, including ‘Black’ British girls [14] as well as Antillean and Creole-Surinamese Dutch girls [13]. (No information available on Cape Verdean Dutch).

Research of Western majority samples show that sexual abuse has a strong association to suicidality [4], and that this relationship also exists among immigrant young female populations in the Netherlands [8]. As Caribbean-Dutch girls on average report their first sexual intercourse at a much younger age than ‘native’ girls [13], this may convey a risk for negative sexual experiences, potentially including sexual abuse.

On the basis of the aforementioned literature, in the present study we expect and explore whether socio-economic factors, household structure, sexual trauma, and emotional and externalizing problems are risk factors for attempted suicide among Dutch girls of Caribbean and Cape Verdean descent.

## Methods

### Study design and procedure

Data were obtained from the YMR, a child and adolescent health surveillance monitor carried out by the Municipal Public Health Service. All YMR data were obtained within routine health examinations which had been ethically approved by the local government previously.

For the present study we used data of 14 to 16 year old students. About 85% of all secondary schools in Rotterdam participated in the YMR. The survey was filled out in the classroom on a voluntary basis between September 2003 and July 2006. Parents received written information on the YMR and could withdraw their child’s participation. The response rate was about 90%.

### Ethnicity

The ethnicity of the youngsters was established by using the country of birth of the father and mother. However, three exceptions existed: Girls with a mixed ethnic background, third generation immigrant girls, and Creole-Surinamese girls (since the Surinamese population consists of a number of ethnic subgroups) could only be identified through ethnic self-identification in the dataset. This was done through the item “Which group do you identify mostly with?” 1.Dutch 2. Surinamese 3. Surinamese/Creole 4. Surinamese/South Asian 5. Antillean or Aruban 6. Moroccan 7. Turkish 8. Cape Verdean 9. Other. Thus, respondents considered to be part of the aforementioned exceptional three cases were categorized as minorities only when youngsters self-identified mostly with the specific minority culture. Third generation immigrant youth concerned 6 Antillean Dutch youth and one Cape Verdean Dutch youth.

### Dependent variable

Life time prevalence of attempted suicide was measured through the following item ‘Have you ever made an attempt to end your life?’[three point scale: never, once or more than once]. In the analyses we dichotomized the answers (Never = no. Once/more than once = yes).

### Independent variables

#### Household structure

Respondents filled out whether they lived with two biological parents in one household, or if they lived in a different household composition. For the analyses, we dichotomized this variable into: Lives not with two biological parents (no versus yes).

#### Emotional problems

Emotional problems were examined with 9 items of a shortened version of the Child Health Questionnaire [15]. The items relate to the presence of certain feelings in the past 4 weeks (e.g. loneliness, pleasure, depressed mood, self-image, anxiety and worrying). Each item is scored on a 5-point likert scale ranging from very often to never. A total sumscore is calculated, which varies between 0 and 100 (a higher score means fewer emotional problems). Chronbach’s alpha of the scale was 0.86.

#### Externalizing problems

Four items about aggression in the past 4 weeks were used [16] (e.g. “have you physically attacked someone?”). Answers were on a 5-point scale ranging from never (0) to very often (4) and showed and alpha of .74. In the analyses the answers were put into three categories 1) never 2) sometimes and 3) frequently.

#### Sexual abuse

Lifetime prevalence of sexual abuse was investigated through asking ‘Have you ever been sexually abused (for instance forced against your will into sexual activities, harassed, raped)’. Never = no. Once/more than once = yes.

#### Socio-economic status and educational track

The postal code of respondents was used as a proxy for socioeconomic class, since no other information was available from the survey regarding this element. Factor scores that link the postal code to socioeconomic class were available from the National Statistics Office. These factor scores are based on a scale of items, e.g., income, hours of work, and educational level. Next, several types of education exist in Dutch secondary school: a ‘vocational track’ (=1) which takes 4 years and where students focus particularly on acquiring vocational skills. The university track (=3) is a 6 year long theoretical program which prepares students for a study at university. The general continued education program (‘middle track’ = 2) prepares students for continued education for professional degrees at college level, and takes 5 years. Students enrolled in vocational tracks often have parents belonging to lower socio-economic strata.

### Statistical analyses

Sample characteristics were obtained using descriptive statistics. Rates of attempted suicide in the minority groups were reported, and their difference with the ‘native’ Dutch group were tested using logistic regression. To examine whether demographics, sexual abuse, emotional and externalizing problems contributed to suicidality in each ethnic group; bivariate (chi-square tests and t-tests) and multivariate analyses (multivariate logistic regression) were conducted. Finally, to examine if differences in rates of attempted suicide between ethnicities remained significant, we controlled for the independent variables step by step. All statistical analyses were performed using the Statistical Package for Social Sciences (SPSS), version 20.0 and two-tailed tests were used with α = 0.05.

## Results

### Sample description

Minority girls were overrepresented in vocational educational tracks and among the lower socio-economic strata, and they disproportionately lived in families that are not composed of two biological parents. Rates of emotional problems and sexual abuse were quite comparable across ethnicities, while aggressive behavior was twice as common among minorities compared to ‘native’s (see Table 1).

**Table 1:** Sample characteristics by ethnicity of female students (*N* = 5611), aged 14–16 in Rotterdam, The Netherlands 2003–2006

	Dutch	Creole-Surinamese	Antillean	Cape- Verdean
	*N* = 4691	*N* = 130	*N* = 357	*N* = 433
	%, mean (sd)^a^	%, mean (sd)^a^	%, mean (sd)^a^	%, mean (sd)^a^
Socio-demographic factors				
Age				
14	56.6%	48.5%	36.4%	37.9%
15	38.4%	36.2%	48.5%	51.3%
16	4.9%	15.4%	15.1%	10.9%
Level of Education				
Vocational Track	43.0%	76.0%	89.5%	82.0%
Middle Track	25.7%	14.7%	6.8%	9.2%
University Track	31.3%	9.3%	3.7%	8.8%
SES score (−2.7–3.8)^b^	0.16 (1.13)	1.59 (1.35)	1.71 (1.12)	2.13 (1.09)
Does not live with 2 biological parents (yes)	22.9%	71.1%	75.2%	55.0%
Trauma				
Sexual abuse (yes)	8.0%	9.2%	11.0%	8.9%
Problems				
Emotional problems (0–100)	73.9 (15.1)	70.7 (18.3)	72.2 (18.2)	73.9 (17.2)
Aggressive behavior				
Never	75.4%	42.3%	40.8%	46.2%
Occasional	21.5%	42.3%	46.2%	42.5%
Frequent	3.1%	15.4%	13.0%	11.4%

Note: *SES* Socio-Economic Status

^a^Based on descriptive statistics

^b^Highest socioeconomic status = −2.7

### Rates of attempted suicide

Nine percent of Dutch girls reports having survived at least one suicide attempt. The rates of Creole-Surinamese and Antillean Dutch girls were about 1.5 times higher (15.4% and 14.0% respectively). Cape Verdean Dutch girls reported less suicidal behavior (8.2%) than ‘native’ girls, albeit not significantly (see Table 2).

**Table 2:** Non-fatal suicidal behavior by ethnicity of female students aged 14–16 in Rotterdam, The Netherlands 2003–2006

	Dutch		Creole-Surinamese		Antillean		Cape-Verdean	
	*N* = 4691		*N* = 130		*N* = 357		*N* = 433	
	N	(%)	N	(%)	N	(%)	N	(%)
Attempted Suicide								
No	4264	(90.9)	110	(84.6)	307	(86.0)	397	(91.7)
Yes	427	(9.1)	20	(15.4)^a^	50	(14.0)^b^	36	(8.3)

^a^Significant difference with Dutch females (the reference group) at level ≤ 0.05

^b^Significant difference with Dutch females (the reference group) at level ≤ 0.01

### Associations between risk indicators and attempted suicide within the Caribbean groups, Cape Verdean Dutch group and majority Dutch group

Table 3 shows bivariate associations between socio-economic class, educational track, household structure, sexual abuse, emotional- and aggression problems to suicide attempts in four ethnic groups. For Dutch ‘native’ girls, all these aforementioned factors constituted a significant risk to suicidal behavior. Sexual abuse and emotional problems emerged as a significant risk for attempting suicide in both Caribbean groups and Cape Verdeans. Frequent aggression was more often found among suicide attempters in all minority groups, however only among Antillean Dutch girls this indicator reached significance.

**Table 3:** The bivariate association between socio-demographic characteristics, household structure, sexual abuse, wellbeing and externalizing problems to suicide attempts among female students in four ethnic groups (*N* = 5611), aged 14–16 in Rotterdam, The Netherlands 2003–2006

	Dutch (*N* = 4691)			Creole-Surinamese (*N* = 130)			Antillean (*N* = 357)			Cape Verdean (*N* = 433)		
	No SA (*N* = 4264)	SA (*N* = 427)	P	No SA (*N* = 110)	SA (*N* = 20)	P	No SA (*N* = 307)	SA (*N* = 50)	P	No SA (*N* = 397)	SA (*N* = 36)	P
Level of Educational, %												
*Low*	40.6	67.1	<.001	75.2	80.0	.77	89.8	87.8	.61	81.6	86.1	.75
*Middle*	26.3	19.7		14.7	15.0		6.9	6.1		9.3	8.3	
*High*	33.1	13.1		10.1	5.0		3.3	6.1		9.1	5.6	
SES score, mean (SD)	.15 (1.12)	.32 (1.23)	.003	1.54 (1.40)	1.84 (1.06)	.37	1.68 (1.13)	1.97 (0.98)	.12	2.12 (1.11)	2.22 (0.81)	.62
Not living with 2 biological parents, %	21.5	36.5	<.001	68.5	85.5	.14	75.7	72.0	.57	53.9	66.7	.14
Sexual abuse, %	6.2	26.5	<.001	5.5	30.0	<.001	7.9	30.0	<.001	6.9	30.6	<.001
Emotional problems, mean (SD)	75.3 (14.1)	60.0 (18.0)	<.001	74.3 (14.8)	51.3 (23.1)	<.001	74.9 (16.5)	55.8 (19.4)	<.001	75.4 (16.2)	58.3 (20.3)	<.001
Aggressive behavior**, %**												
Never	78.0	48.6	<.001	43.6	35.0	.72	42.8	28.6	<.001	47.5	31.4	.19
Occasional	19.6	40.6		41.8	45.0		47.4	38.8		41.4	54.3	
Frequent	2.3	10.8		14.5	20.0		9.8	32.7		11.1	14.3	

Note: *SA* Attempted Suicide, *SES* Socio-Economic Status

Table 4 shows the multivariate analyses of the aforementioned risk indicators of attempted suicide in four ethnic groups. In the majority Dutch group all risk indicators except socio-economic status, demonstrated a significant risk to attempting suicide. Socio-economic status was not a risk indicator for suicidality in minorities. Sexual abuse was a significant risk indicator among Antillean Dutch and Cape Verdean Dutch girls. Fewer emotional problems were significantly related to less suicidal behavior in all three minority groups. Frequent aggression increased the odds for attempting suicide in Antillean Dutch girls.

**Table 4:** Multivariate analyses of risk indicators of suicidal behavior in four different ethnic groups of female students, aged 14–16 in Rotterdam, The Netherlands 2003–2006. (Four separate models)

	Dutch (*N* = 4691)	Creole-Surinamese (*N* = 130)	Antillean (*N* = 357)	Cape Verdean (*N* = 433)
	OR (95% CI)	OR (95% CI)	OR (95% CI)	OR (95% CI)
Level of education				
*Vocational track*	REF	REF	REF	REF
*Middle track*	0.56 (0.42–0.74)^**^	0.38 (0.04–3.75)	0.16 (0.02–1.52)	0.68 (0.17–2.67)
*University track*	0.35 (0.25–0.49)^**^	0.10 (0.00–2.73)	2.18 (0.45–10.48)	0.60 (0.13–2.79)
SES	0.99 (0.90–1.09)	1.39 (0.85–2.29)	1.38 (0.95–2.10)	1.24 (0.83–1.84)
Not living with 2 biological parents	1.44 (1.13–1.84)^**^	0.92 (0.19–4.37)	0.63 (0.26–1.52)	1.21 (0.54–2.70)
Sexual abuse	2.52 (1.87–3.40)^**^	4.00 (0.63–25.65)	4.21 (1.56–11.35)^**^	4.39 (1.79–10.78)^**^
Emotional problems	0.86 (0.85–0.88) ^**^	0.80 (0.71–0.91) ^**^	0.86 (0.81–0.91)^**^	0.88 (0.83–0.93)^**^
Aggressive behavior				
*Never*	REF	REF	REF	REF
*Sometimes*	2.09 (1.63–2.68)^**^	0.63 (0.16–2.47)	1.06 (0.43–2.60)	2.07 (0.88–4.84)
*Frequent*	4.40 (2.81–6.88)^**^	0.58 (0.08–4.11)	4.88 (1.71–13.94)^**^	1.12 (0.29–4.27)
R square of the model (Nagelkerke)	0.26	0.41	0.34	0.22

Note: *SES* Socio-Economic Status

^**^Significant at level ≤ 0.01

### Testing a model of risk indicators of suicidal behavior across majority Dutch, Caribbean and Cape Verdean ethnicity

Table 5 demonstrates differences in suicidality associated with a Caribbean or Cape Verdean ethnicity compared to Dutch ethnicity, when controlling through separate steps for; socioeconomic variables, household structure, sexual abuse, emotional problems and aggression. Creole-Surinamese and Antillean ethnicity showed a significant positive association with suicidal behavior (model 1). However, when controlling for socio-demographics, sexual abuse, emotional problems and externalizing behavior this significant positive association disappears (model 6). Moreover, in model 6 Antillean ethnicity and Cape Verdean ethnicity showed a negative association with suicidal behavior. The change from a risk factor to a protective factor was caused by socio-demographic factors (model 2) and aggression (model 5). Furthermore, when comparing only those girls from all four ethnic groups who were enrolled in vocational educational tracks, levels of attempted suicide were not significantly elevated anymore in the ethnic minority groups compared to the Dutch ‘native’ group (Dutch, 14.2%, Creole-Surinamese 16.3%, Antillean 13.6%, Cape Verdean 8.7%) (not presented in table).

**Table 5:** The association between ethnicity and suicidal behavior controlling for socio-demographics, abuse, internalizing problems and externalizing behavior in female students, aged 14–16 in Rotterdam, The Netherlands 2003–2006

	Suicidal behavior (Model 1)^a^	Suicidal behavior (Model 2)^a^	Suicidal behavior (Model 3)^a^	Suicidal behavior (Model 4)^a^	Suicidal behavior (Model 5)^a^	Suicidal behavior (Model 6)^a^
	OR (95% CI) P	OR (95% CI) P	OR (95% CI) P	OR (95% CI) P	OR (95% CI) P	OR (95% CI) P
Ethnicity						
*Dutch*	REF	REF	REF	REF	REF	REF
*Creole-Surinamese Dutch*	1.82 (1.12–2.95) .02	1.11 (0.67–1.86) .68	1.83 (1.11–3.03) 02	1.51 (0.89–2.58) .13	1.12 (0.68–1.87) .65	0.80 (0.45–1.44) .47
*Antillean Dutch*	1.63 (1.19–2.23) .002	0.73 (0.50–1.06) .10	1.57 (1.14–2.18) .007	1.39 (0.98–1.98) .06	1.00 (0.72–1.39) .99	0.59 (0.39–0.89) .01
*Cape Verdean Dutch*	0.91 (0.64–1.29) .58	0.53 (0.36–0.80) .002	0.90 (0.62–1.29) .56	0.82 (0.56–1.20) .82	0.57 (0.40–0.83) .003	0.41 (0.27–0.64) < .001
Socio-demographic factors						
Level of education						
*Vocational track*		REF				REF
*Middle track*		0.52 (0.40–0.66) < .001				0.55 (0.42–0.72) < .001
*University track*		0.30 (0.22–0.40) < .001				0.38 (0.27–0.51) < .001
Socioeconomic status		1.03 (0.95–1.12) .49				1.04 (0.95–1.14) .37
Not living with 2 biological parents		1.75 (1.43–2.15) < .001				1.37 (1.09–1.71) .006
Trauma						
Sexual abuse			5.54 (4.43–6.92) < .001			2.72 (2.09–3.54) < .001
Internalizing Problems						
Emotional problems				0.85 (0.84–0.87) < .001		0.86 (0.85–0.88) < .001
Externalizing behavior						
Aggressive behavior						
*Never*					REF	REF
*Occasional*					2.95 (2.42–3.60) < .001	1.94 (1.55–2.43) < .001
*Frequent*					6.19 (4.51–8.47) < .001	3.72 (2.56–5.39) < .001

^a^Model 1 only ethnicity; Model 2 ethnicity and socio-demographics; Model 3 ethnicity and trauma; Model 4 ethnicity and internalizing problems; Model 5 ethnicity and externalizing problems; Model 6 all independent variables

## Discussion

To our knowledge, this study is the first to investigate suicide attempts of girls of Caribbean and Cape Verdean descent in mainland Europe (i.e. the Netherlands). The increased rates of attempted suicide of Antillean Dutch girls underpin results of a Dutch report by the Amsterdam Municipal Public Health Services showing a higher 12- months incidence of suicidal ideation (27.8 versus 17.7%) and attempts (5.3 versus 1.8%) among Antillean-Dutch girls in Amsterdam (The Netherlands) compared to ‘native’ girls [17]. This suggests that the vulnerability of Antillean-Dutch girls is not limited to living in Rotterdam. Our results are also in line with a study that showed increased rates of attempted suicide of ‘Black’ young females in the UK [5], and may thus point at a vulnerability to suicidality among young female Caribbean populations across Western Europe. Rates of attempted suicide of Cape-Verdean Dutch girls were much lower than Caribbean as well as ‘native’ Dutch, and self-reported rates of Cape-Verdean immigrant girls residing elsewhere in Europe were unavailable (to the best of our knowledge).

We were unable to retrieve self-reported rates of attempted suicide of Cape Verdean girls living on Cape Verde, and of Antillean and Creole-Surinamese girls living in the Dutch Caribbean. However, self-reported life time rates of suicide attempts (13%) of girls 13 to 18 years old living in other countries in the Caribbean region (e.g. Bahamas and Jamaica) are in quite similar to those of Caribbean immigrant girls in our study (14/15.4%, see [18]. This may suggest that the role of migration to suicidality is modest, yet this would need to be further examined.

Next, important risk indicators such as emotional problems and sexual abuse were associated with an elevated level of attempted suicide in both ‘native’ and Caribbean groups. (although among Creole-Surinamese Dutch this was only visible in the bivariate test). Furthermore, the propensity for aggression increased the risk for attempting suicide of Antillean- and ‘native’ females. Next, girls living in Caribbean- or Cape-Verdean Dutch families not composed of a biological father and mother (highly common for these ethnic groups) were not at heightened risk for suicidal behaviour, while ‘native’ girls in households without two biological parents were more at risk for suicidality. Possibly, the long standing tradition of matrifocalism [9] in Caribbean and African cultures can explain this result.

Once the demographics, sexual abuse, psychological wellbeing and aggression were controlled for, Antillean Ducth had a lower instead of higher risk of suicidal behavior, and the heightened risk in the Creole-Surinamese Dutch group to attempt suicide was no longer observed. This underpins the relevance of socioeconomic class to the epidemiology of suicidal behavior, as pointed out by suicide researchers of the WHO multicenter study in Europe [19]. Suggestions for future research include a larger sample size for Creole Surinamese girls, as well as a longitudinal rather than cross sectional design.

## Conclusion

The present study indicates that the apparently increased propensity to suicidal behavior of Caribbean Dutch girls compared to Dutch ‘native’ girls can be explained by their differences in socio-economic status, education, household structure and increased level of aggressive behavior. Hence, our study underpins the idea that immigrants and their children share certain risk factors with mainstream populations in Europe to the manifestation of suicidal behavior (e.g. sexual abuse and emotional problems as well as they seem to have unique features regarding suicide risk (e.g. no detrimental impact of growing up without biological father in Caribbean immigrant households). Considering the very large proportion of our Caribbean sample that lives in deprived socio-economic circumstances compared to majority Dutch girls, and given that the disproportionate rates of attempted suicide ceased to exist when controlling for these socio-economic aspects, our study sheds a light on the high mental health burden on girls who grow up in poverty and who lack access to higher education. Therefore, our study underpins the need for suicide prevention programs that would target socio-economic and educational disparities in both Caribbean and ‘native’ groups.

## Abbreviation

YMR: Youth Health Monitor, conducted in Rotterdam, The Netherlands
